# Enhanced stiffness in peri-cancerous tissue: a marker of poor prognosis in papillary thyroid carcinoma with lymph node metastasis

**DOI:** 10.1093/oncolo/oyae086

**Published:** 2024-06-21

**Authors:** Lei Hu, Lei Ye, Chong Pei, Chunlei Sun, Chaoxue Zhang, Fan Jiang, Nianan He, Weifu Lv

**Affiliations:** Department of Ultrasound, The First Affiliated Hospital of USTC, Division of Life Sciences and Medicine, University of Science and Technology of People’s Republic of China, Hefei, Anhui 230001, People’s Republic of China; Department of Ultrasound, The First Affiliated Hospital of USTC, Division of Life Sciences and Medicine, University of Science and Technology of People’s Republic of China, Hefei, Anhui 230001, People’s Republic of China; Department of Respiratory and Critical Care Medicine, The First People’s Hospital of Hefei City, The Third Affiliated Hospital of Anhui Medical University, Hefei 230001, People’s Republic of China; Department of Thyroid and Breast Surgery, The First Affiliated Hospital of USTC, University of Science and Technology of People’s Republic of China, Hefei, 230001, People’s Republic of China; Department of Ultrasound, The First Affiliated Hospital of Anhui Medical University, Hefei, Anhui 230001, People’s Republic of China; Department of Ultrasound, The Second Affiliated Hospital of Anhui Medical University, Hefei, Anhui 230001, People’s Republic of China; Department of Ultrasound, The First Affiliated Hospital of USTC, Division of Life Sciences and Medicine, University of Science and Technology of People’s Republic of China, Hefei, Anhui 230001, People’s Republic of China; Department of Radiology, The First Affiliated Hospital of USTC, University of Science and Technology of People’s Republic of China, Hefei 230001, People’s Republic of China

**Keywords:** thyroid cancer, lymph node metastasis, stiffness, peri-cancerous tissue, cancer-associated fibroblasts

## Abstract

**Background:**

The prognostic significance of lymph node metastasis (LNM) in papillary thyroid carcinoma (PTC) remains controversial. Notably, there is evidence suggesting an association between tissue stiffness and the aggressiveness of the disease. We therefore aimed to explore the effect of tissue stiffness on LNM-related invasiveness in PTC patients.

**Method:**

A total of 2492 PTC patients from 3 hospitals were divided into an LNM group and a non-LNM group based on their pathological results. The effects of interior lesion stiffness (E) and peri-cancerous tissue stiffness (E_shell_) on the LNM-related recurrence rate and mortality in each patient with PTC subgroup were analyzed. The activation of cancer-associated fibroblasts (CAFs) and extracellular matrix component type 1 collagen (COL-I) in the lesion were compared and analyzed across different subgroups. The underlying biological basis of differences in each subgroup was identified using RNA sequencing (RNA-seq) data.

**Results:**

The E_shell_ value and E_shell_/E in the LNM group were significantly higher than those in the non-LNM group of patients with PTC (E_shell_: 72.72 ± 5.63 vs 66.05 ± 4.46; E_shell_/E: 1.20 ± 1.72 vs 1.09 ± 1.10, *P *< .001). When E_shell_/E > 1.412 and LNM were both present, the recurrence rate and mortality were significantly increased compared to those of group of patients with LNM (91.67% and 7.29%, respectively). The CAF activation and COL-I content in the E_shell_/E^+^ group were significantly higher than those in the E_shell_/E^−^ group (all *P* < .001), and the RNA-seq results revealed significant extracellular matrix (ECM) remodeling in the LNM-E_shell_/E^+^ group.

**Conclusions:**

Stiff peri-cancerous tissue induced CAF activation, COL-I deposition, and ECM remodeling, resulting in a poor prognosis for PTC patients with LNM.

## Teaser

This study sheds light on the long-standing debate regarding the prognostic impact of lymph node metastasis in patients with papillary thyroid carcinoma.

## Graphical Abstract

**Graphical Abstract:**
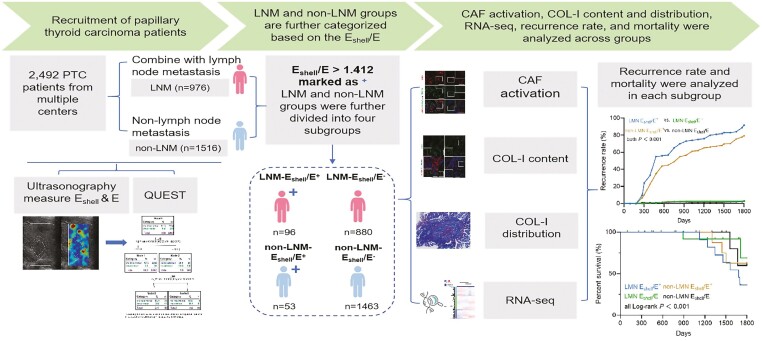

**Implications for practice**

Our study sheds light on the long-standing debate regarding the prognostic impact of LNM in patients with PTC. We demonstrate that peri-cancerous tissue stiffness, particularly when it exceeds a ratio of 1.412, is a clear indicator of poor prognosis in patients with PTC with LNM. This stiffness signals active CAF engagement, collagen deposition, and ECM remodeling. Recognizing these biomechanical changes as prognostic factors offers a new dimension in the preoperative assessment of PTC, enabling clinicians to refine risk stratification and personalize treatment strategies to improve patient outcomes.

## Introduction

Papillary thyroid carcinoma (PTC) is the most common pathological type of thyroid malignancy, accounting for 90% of all thyroid malignancies.^1^ Patients with PTC with non-metastatic thyroid cancer can be successfully treated through surgery, radioactive iodine-131 therapy, and thyroid-stimulating hormone suppression therapy. However, cervical lymph node metastasis (LNM) occurs in approximately 50%-60% of patients with thyroid cancer and is widely considered one of the most important risk factors for the possibility of distant metastasis and a poor prognosis for PTC patients.^2-5^ The association between LNM and the postoperative recurrence rate and mortality of patients with PTC remains controversial. In terms of the LNM-related postoperative recurrence rate and mortality in patients with PTC, some studies have shown significant associations,^2-5^ while others have reported none.^6-9^ To minimize uncertain LNM-related recurrence and mortality in the clinic, overtreatment of PTC is common when LNM is present, but this inevitably increases the risk of treatment-associated complications.^9^ Previous studies on postoperative LNM-related recurrence and mortality in patients with PTC have yielded inconsistent results, which may be the result of other decision-making factors. Thus, the prognosis of patients with PTC cannot be solely predicted based on the presence or absence of LNM.

Biomechanical studies have established a connection between lesion stiffness and the biological behavior and prognosis of patients.^10,11^ It has been proven that the ultrasound elastography technique, which is used to measure the stiffness of thyroid lesions for the differential diagnosis between benign and malignant types, is an accurate and has good diagnostic value.^12-16^ Cancer-associated fibroblasts (CAFs), representing a heterogeneous population of fibroblasts that are recruited and activated to augment tumor progression in many different solid tumors, are the starting point for increased tissue stiffness.^17,18^ In addition to stimulating cancer cell proliferation, angiogenesis, invasion, and metastasis, CAFs also drive tumorigenesis by upregulating the production of extracellular matrix (ECM) components, including type 1 collagen (COL-I).^18^ COL-I is the most abundant ECM scaffolding protein. Its increased deposition in the tumor microenvironment is associated with tumor progression.^17,18^ The increased incidence of metastasis^18^ and drug resistance^19^ has been observed in various human cancers. Several lines of research indicate that the infiltration of CAFs and collagen deposition in the ECM significantly reduces overall survival and disease-free survival in patients with PTC.^20^ Researchers have further demonstrated that the presence of fibrosis is significantly associated with the morphological parameters of invasion and a higher incidence of LNM in all types of thyroid cancer patients. In contrast, the absence of a fibrotic ECM has been found to be indicative of non-invasive and non-metastasizing thyroid cancers.^17,20^ Therefore, an increased CAF activation and fibrosis in the ECM of thyroid cancers can prove valuable in clinical practice for predicting cancer aggressiveness, progression, and poor clinical outcomes.

Herein, we hypothesize that CAF-induced stiffness in both the interior and peri-cancerous regions plays a critical role in influencing the risk of the LNM-related recurrence rate and mortality of patients with PTC. Different tissue stiffness states can be used to differentiate patients with PTC with LNM into 2 fundamentally different risk categories. We tested and explored our hypothesis by retrospectively analyzing differences in interior and peri-cancerous stiffness, CAF activation, and COL-I content in multicenter patients with PTC with and without LNM. Additionally, we further analyzed the underlying biological basis of differences using RNA sequencing (RNA-seq).

## Patients and methods

### Patients

**Figure 1.**
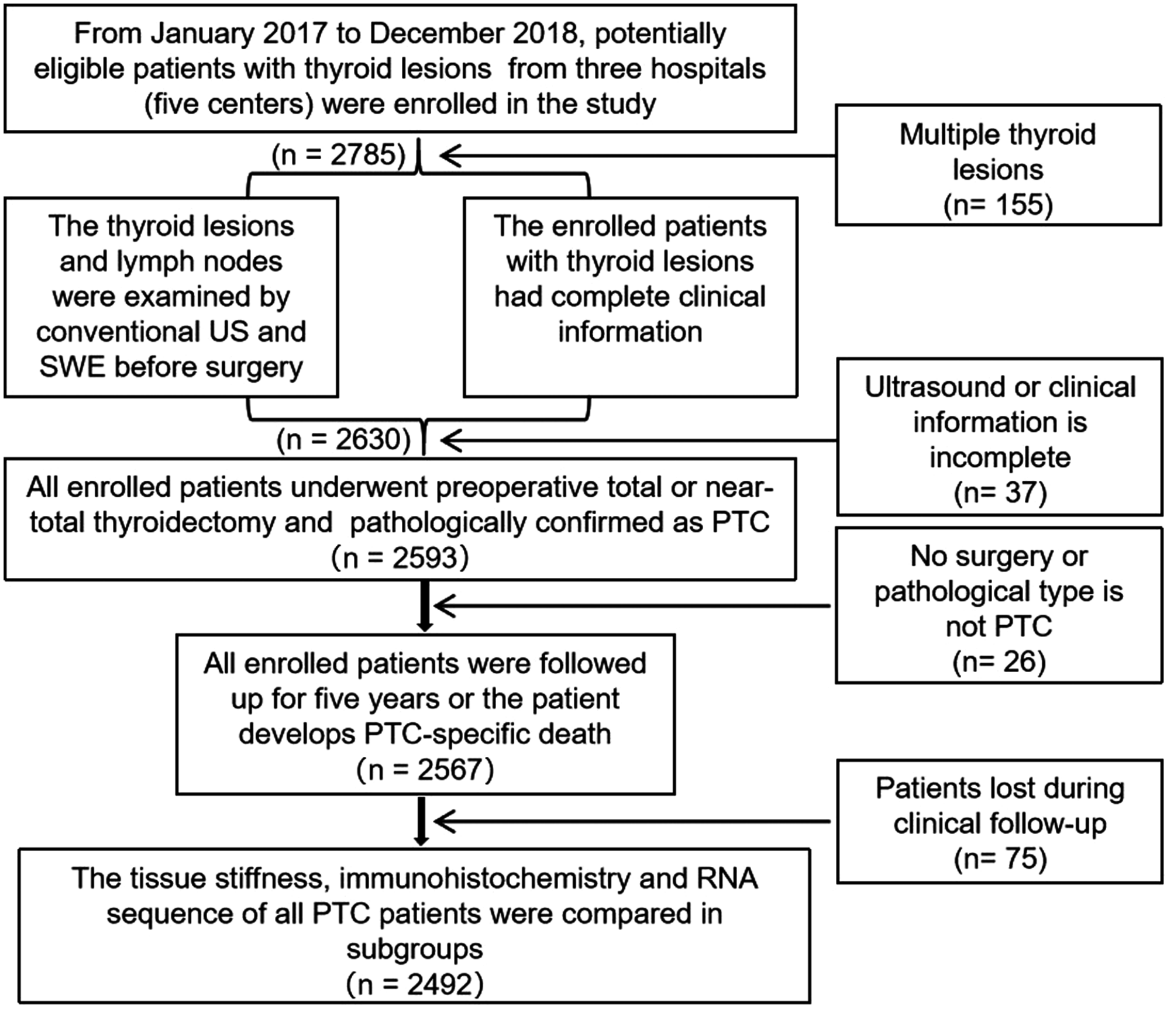
Flowchart of participant selection. Abbreviations: PTC, papillary thyroid carcinoma; SWE, shear wave elastography.

From January 2017 to December 2018, a total of 2492 patients with PTC (2015 women and 623 men) from 3 hospitals (5 centers) were included in the study. This multicenter study was retrospective; the data used was obtained from medical records approved by each participating center. All enrolled patients underwent total or near-total thyroidectomy followed by histopathological examination. Subsequently, the enrolled patients with PTC were divided into an LNM group and a non-LNM group based on the pathological findings related to cervical LNM. Prior to surgery, thyroid lesions and lymph nodes were examined using conventional ultrasonography and shear wave elastography (SWE). Recurrence of PTC refers to recurrent disease based on cytologic, histopathologic, and radiographic criteria.^12,13^ Disease-specific mortality was defined as patient death caused by PTC.^12,13^ All enrolled patients were monitored for a period of 5 years, and the clinical follow-up duration extended from the initial thyroidectomy to the discovery of disease presence (for recurrence analyses), the PTC-specific death of the patient (for mortality analyses), or the latest clinical visit for patients with no disease-free survival. The patient recruitment flow chart is shown in Figure 1.

### Acquisition of the stiffness of thyroid cancer, peri-cancerous, and cervical lymph node tissues

**Figure 2.**
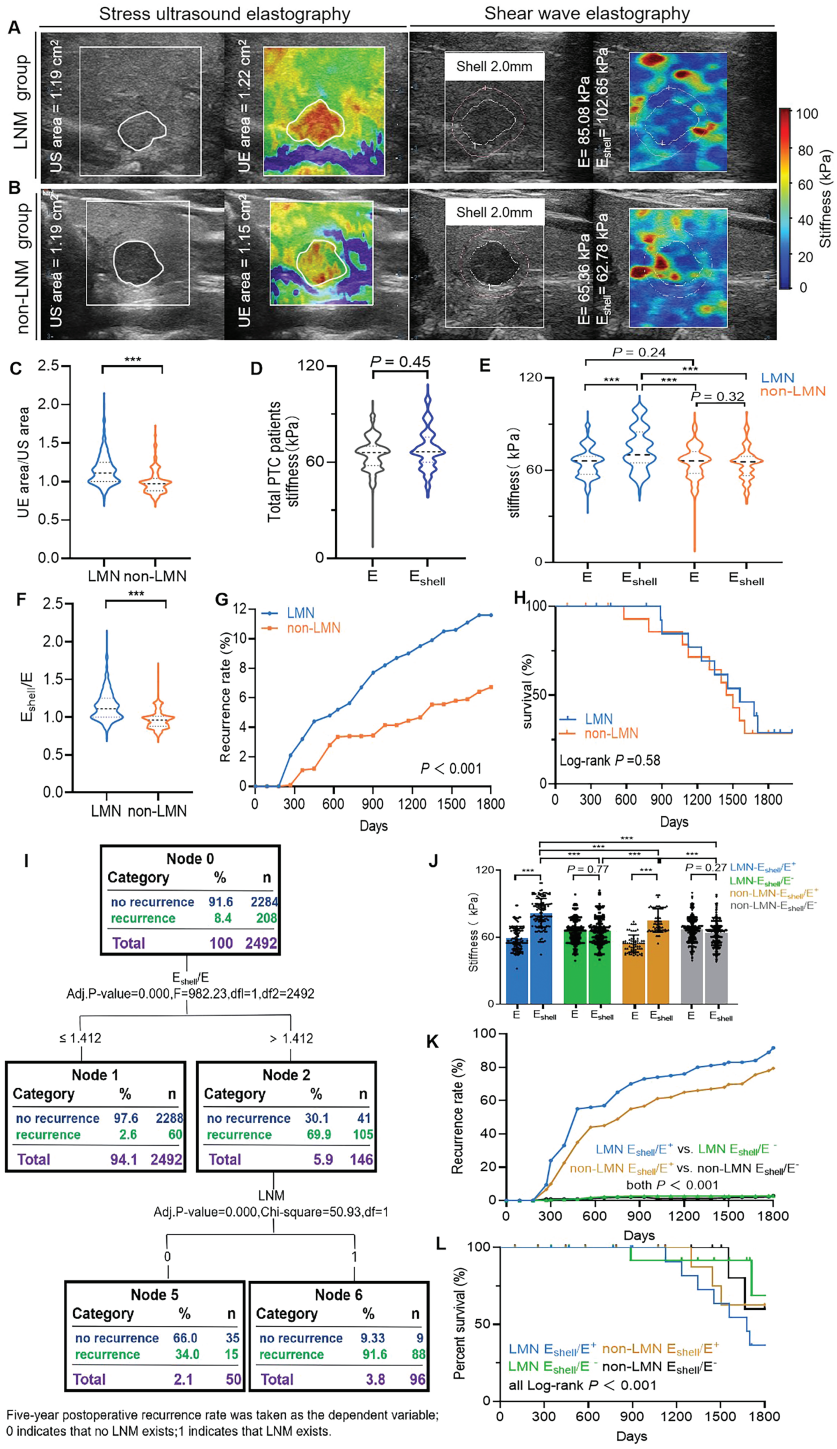
Different tissue stiffness states affect LNM-related invasiveness in patients with PTC. (A) Stress UE and SWE image of the lesions in the non-LNM group patients with PTC. Left image: stress UE indicated that the stiffness of the lesions mainly existed in the interior of the lesion; UE area/US area = 1.03 (white circle). Right image: SWE displays an E value of 65.36 kPa, E_shell_ value of 62.78 kPa, and E_shell_/E = 0.96. (B) Stress UE and SWE image of the lesions in LNM group patients with PTC. Left image: stress UE indicated that the stiffness range of the lesions was larger than that inside the lesions; UE area/US area = 0.97 (white circle). Right image: SWE displays an E value of 85.08 kPa, E_shell_ value of 102.65 kPa, and E_shell_/E = 1.21. (C) Quantification of UE area/US area. UE area/US area in the LNM group was significantly higher than that in the non-LNM group (1.15 ± 0.81 vs 0.99 ± 0.22, *P* < .001). (D) Quantification of tissue stiffness in all patients with PTC. The E and E_shell_ values of all patients with PTC exhibited no statistical differences (E 64.30 ± 2.81 kPa vs E_shell_ 65.89 ± 3.54 kPa, *P* = .45). (E) Quantification of tissue stiffness in the LNM and non-LNM groups. The E_shell_ value was significantly higher than the E value in LNM group patients with PTC (E 63.85 ± 4.22 kPa vs E_shell_ 72.72 ± 5.63 kPa, *P* < .001). There was no statistical difference between the E and E_shell_ values in the non-LNM group (E 64.98 ± 3.43 vs E_shell_ 65.05 ± 4.46 kPa, *P* < .32). The E_shell_ value of the LNM group was significantly higher than that of the non-LNM group (LNM 72.72 ± 5.63 kPa vs non-LNM 64.98 ± 3.43 kPa, *P* < .001). There was no significant difference in the E value between the LNM and non-LNM groups (LNM 63.85 ± 4.22 kPa vs non-LNM 64.98 ± 3.43 kPa, *P* = .24). (F) Quantification of E_shell_/E in the LNM and non-LNM groups. The E_shell_/E of the LNM group was significantly higher than that of the non-LNM group (LNM 1.20 ± 1.72 vs non-LNM 1.09 ± 1.10, *P* < .001). (G) The 5-year postoperative recurrence rate of patients with PTC in the LNM group was significantly higher than that in the non-LNM group (LNM 11.61% vs non-LNM 6.27%, *P* < .001). (H) The 5-year postoperative mortality of patients with PTC in the LNM group had no significant difference compared with that in the non-LNM group (LNM 0.92% vs non-LNM 0.86%, *P* = .58). (I) The effects of E, E_shell_, and E_shell_/E on LNM-related invasiveness in patients with PTC were analyzed using QUEST. The 5-year postoperative recurrence rate was taken as the dependent variable when the E_shell_/E ratio was > 1.412, the 5-year postoperative recurrence rate of patients with PTC was 69.9% (node 2), and the recurrence probability increased to 91.3% (node 6) when LNM was present. (J) Quantification of the E and E_shell_ values of 4 subgroups. The E_shell_ value of LNM-E_shell_/E^+^ group patients with was significantly higher than the E value, and the E_shell_ value was higher than that of the other 3 subgroups (all *P* < .001). The E_shell_ value of non-LNM-E_shell_/E^+^ group patients with PTC was significantly higher than the E value, and the E_shell_ value was higher than that in 2 E_shell_/E^−^ groups (all *P* < .001). There was no statistical difference between the E and E_shell_ values of the LNM-E_shell_/E^−^ and non-LNM-E_shell_/E^−^ groups (LNM-E_shell_/E^−^, *P* = .77; non-LNM-E_shell_/E^−^, *P* = .27). (K) The 5-year postoperative recurrence rate of PTC patients in 4 subgroups. LNM-E_shell_/E^+^, 91.67%; LNM-E_shell_/E^−^, 2.84%; non-LNM-E_shell_/E^+^, 79.41%; non-LNM-E_shell_/E^−^, 2.83% (all *P* < .001). (L) The 5-year postoperative mortality of patients with PTC in 4 subgroups. LNM-E_shell_/E^+^, 7.29%; LNM-E_shell_/E^−^, 0.22%; non-LNM-E_shell_/E^+^, 3.77%; non-LNM-E_shell_/E^−^, 0.75% (all *P* < .001). In all panels, ***P < .001. Blue indicates the LNM-E_shell_/E^+^ group, *n* = 96. Green indicates the LNM-E_shell_/E^−^ group, *n* = 880. Brown indicates the non-LNM-E_shell_/E^+^ group, *n* = 53. Black indicates the non-LNM-E_shell_/E^−^ group, *n* = 1463. LNM, lymph node metastasis; PTC, papillary thyroid carcinoma; SWE, shear wave elastography; QUEST = quick, unbiased, efficient statistical tree; UE, ultrasound elastography.

The stiffness of thyroid cancer tissue (E), peri-cancerous tissue (E_shell_), and cervical lymph node tissue was measured using a Mindray Resona 7 ultrasonography diagnostic system (Mindray Medical) equipped with the 11L3 transducer, along with SWE and Shell measurement software. The long axis of the thyroid lobe containing the thyroid lesion was selected for sampling. Then, stress ultrasound elastography images of the thyroid lesion were captured. Subsequently, the ratio between the area of the thyroid lesion obtained by grayscale ultrasound (US area) and that obtained by stress ultrasound elastography (UE area) was determined (Figure 2A). Following this, the boundary of the thyroid lesion was outlined using a tracing method, and the E value was measured using SWE. Finally, based on the previously outlined thyroid lesions, the stiffness of peri-cancerous tissue was measured by expanding 2 mm outward from the lesion’s outline using the Shell function, and the result was recorded as E_shell_ (Figure 2B). When suspicious cervical lymph nodes were present, tissue stiffness was assessed using SWE. Normal cervical lymph node stiffness, defined by preoperative ultrasound as suspicious for metastasis but confirmed to be negative through tissue biopsy or postoperative pathology, was used as a control. After surgery and clinical follow-up, the recurrence rate, mortality, E value, E_shell_ value, stiffness of the cervical lymph node, and clinical characteristics of each subgroup of patients with PTC were analyzed. The pooled data were used to analyze the relationship between tissue stiffness and the post-operative LNM-related recurrence rate and mortality in patients with PTC.

### Classification tree analysis for LNM-related aggressiveness

To analyze the influence of thyroid cancer and peri-cancerous tissue stiffness on LNM-related aggressiveness in patients with PTC, we used the Quick, Unbiased, Efficient Statistical Tree (QUEST) method was used to evaluate the prognostic effects of E, E_shell_, and E_shell_/E on LNM-related invasiveness in patients with PTC.^16^

#### Data preparation

The dataset comprised measurements of E, E_shell_, E_shell_/E, and the presence of LNM in patients with PTC. The 5-year postoperative recurrence rate served as the dependent variable, reflecting the disease’s aggressiveness

#### Building the classification tree

The QUEST method was used to construct a classification tree that predicts recurrence likelihood based on the independent variables E, E_shell_, E_shell_/E, and LNM. The minimal parent and child node sizes were set to 10 and 5, respectively, ensuring that each split in the tree resulted in at least 10 observations in the parent node and at least 5 observations in each child node.

#### Model validation

The robustness of the classification tree was evaluated using 10-fold cross-validation. The dataset was divided into 10 subsets, with 9 subsets used for training the model and the remaining subset for testing. This process was repeated 10 times, ensuring that each subset was used for testing once. The resulting classification tree can be used to identify patterns and thresholds in the stiffness measurements that are associated with a higher risk of recurrence.

### Detection of CAF activation and collagen deposition in tissue samples from patients with PTC

All patients underwent thyroidectomy in the surgery department of their respective hospitals. Cancer tissue samples from 62 enrolled PTC patients (30 LNM patients and 32 non-LNM patients) were analyzed. To examine variations in CAF activation and collagen deposition among subgroups of patients with PTC, multiplex immunofluorescence (mIF) staining (1:100; Absin; abs50014-20T) was conducted to detect the CAF marker platelet-derived growth factor receptor alpha (PDGFRα), alpha-smooth muscle actin (αSMA), the myofibroblast marker, and phospho-myosin light chain 2 (p-MLC2) for actomyosin contractility, as well as the ECM component COL-I. The following primary antibodies were incubated overnight at 4 °C: αSMA (1:400; Sigma-Aldrich; A2547), PDGFRα (1:100; Cell Signaling; 3174), p-MLC2 (1:100; Cell Signaling; 3671T), and COL-I (1:100; Abcam; ab34710). Masson’s trichrome staining (1:100; Ebiogo; B022-07242311) was used to analyze the distribution of collagen fibers in the whole slide imaging (WSI) sections of PTC subgroup patients. Patient data and samples were collected in a prospective database and analyzed retrospectively. Quantification of all staining procedures was performed using the NIH ImageJ 1.51s analysis software, with a consistent threshold applied for each stain. Positivity was assessed in the field of view per sample. Imaging was conducted using an LSM 510 META laser scanning microscope (Zeiss).

### RNA sequencing and data analysis

To identify gene expression signatures associated with tissue stiffness and differentiate LNM-related invasiveness, RNA-seq was performed on each group of PTC patients. Total RNA was isolated using a mini RNA isolation kit (Qiagen; 74106). Then, libraries with different indexes were multiplexed and loaded on an Illumina HiSeq/Illumina NovaSeq/MGI2000 instrument for RNA-seq using a 2 × 150 paired-end configuration according to the manufacturer’s instructions. Quality was assessed using a Hisat2 (v2.0.1) Bioanalyzer. RNA-seq libraries were prepared according to the manufacturer’s protocol.

The screening criteria for differentially expressed genes were as follows: basemean >10, padj <0.01, and log2 (fold change) >1. Subsequently, the first 1500 upregulated differentially expressed genes were imported into the DAVID database (https://david.ncifcrf.gov/) for pathway enrichment analysis. Through Gene Ontology (GO) and Kyoto Encyclopedia of Genes and Genomes (KEGG) analysis, differentially expressed genes in the tissue samples of each group of patients with PTC were investigated, with a focus on pathways related to cell proliferation, cell migration, the interactions between cells and ECM, and ECM fibrosis.

### Tissue stiffness, CAF activation, and Ki-67 expression were detected in the metastatic lymph nodes and normal lymph nodes of patients with PTC

To further examine whether differences in the lesion samples of patients with PTC affect the LNM-related metastasis rate and mortality, metastatic lymph nodes, and normal lymph nodes were further compared. A total of 10 metastatic and 10 normal lymph nodes from enrolled patients with PTC were analyzed. To detect CAF activation in the metastatic lymph nodes and normal lymph nodes of PTC patients, mIF staining (1:100; Absin; abs50014-20T) was performed. The antibodies used included αSMA (1:400; Sigma-Aldrich; A2547), PDGFRα (1:100; Cell Signaling; 3174), and p-MLC2. Quantification of the percentage of the positive area expressing Ki-67 (1:100; Cell Signaling; D3B53) was conducted based on immunohistochemical (IHC) staining to determine the proliferative activity of cells in all lymph nodes.

## Statistical analyses

All statistical analyses were conducted using GraphPad Prism 8.0 software (GraphPad Software) and the Statistical Package for Social Science version 22.0 (SPSS) (IBM SPSS, Inc.). Quantitative data are presented as the mean ± SD. Qualitative data are presented as frequencies. Categorical variables were compared using the *χ*^2^ test and Fisher’s exact probability test. Comparison of the median and interquartile range of continuous variables in nonparametric independent samples was performed using the Wilcoxon-Mann-Whitney test. Kaplan-Meier analysis was used to estimate the survival probability, and the log-rank test was used to compare the differences between the Kaplan-Meier curves of patients in various subgroups. The QUEST method was used to classify and analyze the prognostic effects of E and E_shell_ on patients with PTC with LNM. All *P*-values were 2 sided, with a value <.05 considered statistically significant.

## Results

### Clinicopathological characteristics and tissue stiffness of PTC patients between LNM and non-LNM groups

The demographic characteristics, clinicopathological characteristics, and E and E_shell_ values of the 2492 patients with PTC in this study are presented in Table 1. There were no significant differences in gender, age, or lesion size between the LNM and non-LNM groups. However, compared with the non-LNM group, the LNM group had a higher proportion of patients who received radioactive iodine treatment and patients who were in stage III or IV (both *P* < .001). Through stress ultrasound elastography measurements, it was determined that the UE area/US area in the LNM group was significantly higher than that in the non-LNM group (1.15 ± 0.81 vs 0.99 ± 0.22, *P* < .001, Figure 2C). Although there was no statistical difference in the E value between the LNM and non-LNM groups, the E_shell_ value and E_shell_/E in the LNM group were significantly higher than in the non-LNM group [E_shell_: (72.72 ± 5.63 vs 66.05 ± 4.46) kPa], [E_shell_/E: 1.20 ± 2.7 vs 1.09 ± 1.1, both *P *< .001; Figure 2D-F]. The 5-year postoperative recurrence rate of patients with PTC in the LNM group was significantly higher than that in the non-LNM group [113/976 (11.61%) vs 95/1516 (6.27%), *P* < .001; Figure 2G]. However, there was no significant difference in the 5-year postoperative mortality between patients in the LNM group and those in the non-LNM group (*P* = .87; Figure 2H, Table 1).

**Table 1.** The demographic characteristics, clinicopathological characteristics, and tissue stiffness of all patients with PTC.

		Total (*n*%)	LNM (*n*%)	Non-LNM (*n*%)	*p*
Medical centers					
Center 1	The 1st Affiliated Hospital of University of Science and Technology of People’s Republic of China	648/2492 (26.00)	268/648 (41.35)	380/648 (58.64)	<.001
Center 2	The 1st Affiliated Hospital of University of Science and Technology of People’s Republic of China (South Campus)	348/2492 (13.96)	184/348 (52.87)	164/348 (47.12)	< 0.001
Center 3	The 1st Affiliated Hospital of Anhui Medical University	527/2492 (21.14)	196/527 (37.19)	331/527 (62.80)	<.001
Center 4	The 1st Affiliated Hospital of Anhui Medical University (South Campus)	339/2492 (13.60)	113/339 (33.33)	226/339 (66.67)	<.001
Center 5	The 2nd Affiliated Hospital of Anhui Medical University	630/2492 (25.28)	215/630 (34.12)	415/630 (65.87)	<.001
Total (*n*%)		—	976/2492 (39.17)	1516/2492 (60.83)	<.001
Sex (female%)		1785/2492 (71.6)	702/976 (71.9)	1083/1516 (71.4)	.79
Median age (IQR) (years)		46 (21-74)	47 (21-64)	51 (27-74)	.33
Age ≥ 60 years (*n*%)		889/2492 (35.67)	334/976 (34.22)	555/1516 (36.60)	.22
Median tumor size (IQR), mm		17.8 (11.2-21.4)	16.8 (11.2-19.7)	17.7 (12.3-21.9)	.24
Tumor size > 10 mm (*n*%)		1,325/2,492 (53.17)	524/976 (53.69)	801/1,516 (52.83)	.68
Tumor stage III/IV (*n*%)		19 (0.76)	10 (1.02)	9 (0.59)	.44
*BRAF* ^V600E^ mutation (*n*%)		2118/2492 (85.0)	828/976 (84.83)	1290/1516 (85.09)	.86
^131^I treatment (*n*%)		811/2,492 (32.5)	315/976 (32.3)	55/1516 (3.63)	<.001
Median follow-up time (months)		49 (24-60)	48 (24-60)	55 (50-60)	.12
UE area/US area		1.01 ± 0.61	1.15 ± 0.81	0.99 ± 0.22	<.001
E (mean ± SD, kPa)		64.30 ± 2.81	63.85 ± 4.22	64.98 ± 3.43	.24
E_shell_ (mean ± SD, kPa)		67.89 ± 3.54	72.72 ± 5.63	65.05 ± 4.46	< 0.001
E_shell_/E (mean ± SD)		1.12 ± 1.31	1.20 ± 1.72	1.09 ± 1.10	< 0.001
Recurrence rate (*n*%)		208/2492 (8.35)	113/976 (11.61)	95/1,516 (6.27)	< 0.001
Mortality (*n*%)		22/2492 (0.88)	9/976 (0.92)	13/1516 (0.86)	0.58

Abbreviations. E, interior lesion stiffness of patient with PTC; E_shell_, peri-cancerous tissue stiffness; LNM, lymph node metastasis; PTC, papillary thyroid carcinoma; UE area, the area of stress ultrasound elastography; US area, the area of the thyroid lesion in gray scale ultrasound. Recurrence rate and mortality refer to those 5 years after surgery.

### Effects of tissue stiffness on LNM-related invasiveness in patients with PTC

According to the results of the QUEST classification tree algorithm, the E and E_shell_ values were not included in the algorithm because they did not improve the accuracy of the algorithm for predicting the probability of metastasis in PTC patients. If the E_shell_/E ratio was ≤ 1.412, the probability of a 5-year postoperative recurrence rate in PTC patients was only 2.6%, independent of the presence or absence of LNM (node 1). If the E_shell_/E ratio was > 1.412, the 5-year postoperative recurrence rate in PTC patients was 69.9% (node 2), and the LNM was considered. When combined LNM was present, the probability of recurrence increased to 91.6% (node 6) (Figure 2I).

Using E_shell_/E ≤ 1.412 as the cutoff value, cases where E_shell_/E > 1.412 were marked as E_shell_/E^+^, and the rest were marked as E_shell_/E^−^. Patients with PTC in the LNM and non-LNM groups were further divided into 4 subgroups: LNM-E_shell_/E^+^, LNM-E_shell_/E^−^, non-LNM-E_shell_/E^+^, and non-LNM-E_shell_/E^−^. The E_shell_ value of patients with PTC in the LNM-E_shell_/E^+^ group was significantly higher than the E value, and their E_shell_ value was higher than that in the other 3 subgroups (all *P* < .001; Figure 2J). The 5-year postoperative recurrence rate in the LNM-E_shell_/E^+^ group increased to 91.67% compared with that in the original LNM group, and the mortality increased to 7.29%. In contrast, the 5-year postoperative recurrence rate (2.84%) and mortality (0.22%) of patients with PTC in the LNM-E_shell_/E^−^ group were significantly lower than those in the original LNM group (*P* < .001). Compared with those in the original non-LNM group, the 5-year postoperative recurrence rate in the non-LNM-E_shell_/E^+^ group increased to 79.41%, and the mortality increased to 4.41% (Figure 2K). In contrast, the non-LNM-E_shell_/E^−^ group exhibited a decrease in the 5-year postoperative recurrence rate to 2.83% and a decrease in mortality to 0.69% compared with the original non-LNM group (Figure 2L). The clinical characteristics of the 4 subgroups are presented in Table 2.

**Table 2.** Clinical characteristics of PTC patients grouped by LNM combined with tissue stiffness status.

Characteristics	LNM			Non-LNM		
	E_shell_/E^+^	E_shell_/E^−^	*P*	E_shell_/E^+^	E_shell_/E^−^	*P*
*n* (%)	96/976 (9.83)	880/976 (90.16)	<.001	68/1,516 (4.49)	1,448/1,516 (95.51)	<.001
Median (IQR), years	44 (21-55)	49 (33-67)	.44	44 (27-64)	57 (35-74)	.21
Age ≥ 60 years (%)	22/96 (22.92)	312/880 (35.45)	.01	16/68 (23.52)	539/1,448 (37.22)	.32
Median tumor size (IQR), mm	15.8	17.1	.44	16.7	18.8	.21
Tumor size > 10 mm (*n*%)	45/96 (46.89)	479/880 (54.43)	.16	26/68 (38.23)	775/1,448 (53.52)	.56
*BRAF* ^V600E^ mutation (*n*%)	88/96 (91.67)	740/880 (84.09)	<.001	45/68 (66.17)	1,245/1,448 (85.98)	.96
Tumor stage III/IV (*n*)	8	2	<.001	5	4	<.001
^131^I treatment (%)	33/96 (34.38)	282/880 (32.45)	.643	5/68 (7.35)	50/1,448 (3.45)	<.001
Follow-up time (months)	47 (24-60)	58 (55-59)	.24	58 (55-60)	57 (56-60)	.15
E (mean ± SD, kPa)	63.85 ± 7.21	64.15 ± 4.22	.33	65.75 ± 9.43	64.05 ± 7.24	.34
E_shell_ (mean ± SD, kPa)	84.72 ± 7.67	64.72 ± 8.67	<.001	74.72 ± 8.56	63.72 ± 9.65	<.001
E_shell_/E (mean ± SD)	1.62 ± 1.18	1.04 ± 0.78	<.001	1.13 ± 0.98	0.98 ± 0.44	<.001
Recurrence rate (%)	88/96(91.67)	25/880 (2.84)	<.001	54/68 (79.41)	41/1,448 (2.83)	<.001
Mortality risk(%)	7/96 (7.29)	2/880 (0.22)	<.001	3/68 (4.41)	10/1,448 (0.69)	<.001

### Highly activated CAFs in 2 E_shell_/E^+^ subgroups of patients with PTC

**Figure 3.**
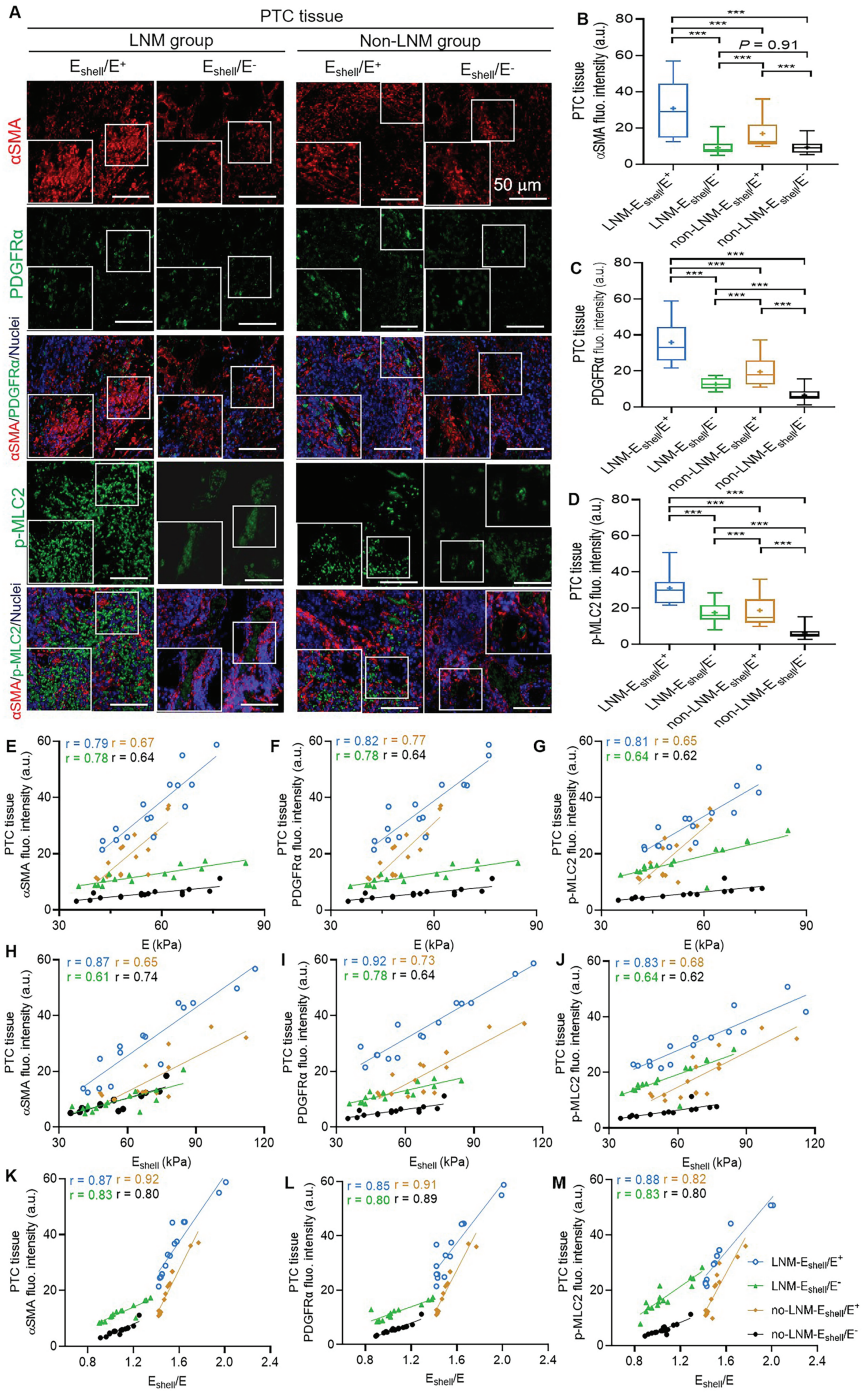
CAFs are highly activated in the LNM-E_shell_/E^+^ group compared with the other 3 subgroups of patients with PTC and positively associated with tissue stiffness in 4 subgroups of patients with PTC. (A) mIF staining of αSMA (red), PDGFRα (green), and p-MLC2 (green) on paraffin sections of the 4 subgroups. Quantification of (B) αSMA fluorescence intensity, (C) PDGFRα fluorescence intensity, and (D) p-MLC2 fluorescence intensity in 4 subgroups. The expression of αSMA, PDGFRα, and p-MLC2 in the LNM-E_shell_/E^+^ group was significantly higher than that in the other 3 subgroups, and the CAF activation of the non-LNM-E_shell_/E^+^ group was significantly higher than that of the LNM-E_shell_/E^−^ and non-LNM-E_shell_/E^−^ groups (all *P* < .001). Spearman’s correlation of the E value with the fluorescent intensity of αSMA (E), PDGFRα (F), and p-MLC2 (G) based on mIF staining in 4 subgroups of patients with PTC (all subgroups *r* ≥ 0.60, all *P* < .001). Spearman’s correlation of the E_shell_ value with fluorescent intensity of αSMA (H), PDGFRα (I), and p-MLC2 (J) based on mIF staining in 4 subgroups of patients with PTC (all subgroups *r* ≥ 0.60, all *P* < .001). Spearman’s correlation of E_shell_/E with fluorescent intensity of αSMA (K), PDGFRα (L), and p-MLC2 (M) based on mIF staining in 4 subgroups of patients with PTC (all subgroups *r* ≥ 0.80, all *P* < .001). In all panels, ***P < .001. Blue indicates the LNM-E_shell_/E^+^ group, *n* = 15. Green indicates the LNM-E_shell_/E^−^ group, *n* = 15. Brown indicates the non-LNM-E_shell_/E^+^ group, *n* = 16. Black indicates the non-LNM-E_shell_/E^−^ group, *n* = 16. Scale bars = 50 µm. Abbreviations: αSMA, alpha-smooth muscle actin; au, arbitrary unit; CAFs, cancer-associated fibroblasts; LNM, lymph node metastasis; PTC, papillary thyroid carcinoma; PDGFRα, platelet-derived growth factor receptor alpha; p-MLC2, phospho-myosin light chain 2; mIF, multiplex immunofluorescence.

The clinical characteristics and tissue stiffness information of 30 LNM patients (15 LNM-E_shell_/E^+^ vs 15 LNM-E_shell_/E) and 32 non-LNM patients (16 non-LNM-E_shell_/E^+^ vs 16 non-LNM-E_shell_/E^−^) included in the analysis of CAF activation, collagen content, and their distribution are depicted in Table 3. The mIF fluorescence intensity of the expression of αSMA, PDGFRα, and p-MLC2 in the LNM-E_shell_/E^+^ group was significantly higher than that in the other 3 subgroups. Additionally, the CAF activation in the non-LNM-E_shell_/E^+^ group was significantly higher than that in the LNM-E_shell_/E^−^ and non-LNM-E_shell_/E^−^ groups (Figure 3A-D, all *P* < .001). These findings indicate high CAF activation and ECM remodeling in the E_shell_/E^+^ group, especially in the LNM-E_shell_/E^+^ subgroup.

**Table 3.** Clinical characteristics of PTC patients were detected by CAF activation and collagen deposition.

Characteristics	LNM (*n* = 30)			Non-LNM (*n* = 32)		
	E_shell_/E^+^ (*n* = 15)	E_shell_/E^−^ (*n* = 15)	*p*	E_shell_/E^+^ (*n* = 16)	E_shell_/E^−^ (*n* = 16)	*p*
Sex (female *n*%)	8/15 (53.33)	8/15 (53.33)	1.00	9/16 (56.25)	9/16 (56.25)	1.00
Median age (IQR), years	43 (21-50)	44 (34-60)	.44	44 (26-64)	55 (35-66)	.21
Age ≥ 60 years (*n*%)	7/15 (46.67)	7/15 (46.67)	1.00	5/16 (31.25)	5/16 (31.25)	1.00
Median tumor size (IQR), mm	15.8 (11.2-19.5)	17.12 (12.2-21.3)	.44	16.7 (13.2-19.4)	18.8 (12.5-21.2)	.21
Tumor size > 10 mm (*n*%)	5/15 (33.33)	5/15 (33.33)	1.00	8/16 (50.00)	8/16 (50.00)	1.00
Tumor stage III/IV (*n*%)	5/15 (33.33)	2/15 (13.33)	.39	2/16 (12.50)	0/16 (0)	.48
*BRAF* ^V600E^ mutation (*n*%)	14/15 (93.33)	14/15 (93.33)	1.00	14/16 (87.50)	14/16 (87.50)	1.00
^131^I treatment (*n*%)	7/15 (46.67)	5/15 (33.33)	.71	2/16 (12.50)	2/16 (12.50)	1.00
Median follow-up time (months)	47 (24-60)	58 (55-59)	.24	58 (55-60)	57 (56-60)	.15
E (mean ± SD, kPa)	63.85 ± 4.22	63.33 ± 5.23	.33	64.15 ± 3.44	64.25 ± 3.35	.21
E_shell_ (mean ± SD, kPa)	89.72 ± 4.59	69.88 ± 2.68	<.001	77.72 ± 4.57	64.72 ± 3.66	<.001
E_shell_/E (mean ± SD)	1.64 ± 1.19	1.01 ± 0.25	<.001	1.57	0.99	<.001
Recurrence rate (*n*%)	14/15 (93.33)	2/15 (13.33)	<.01	4/16 (25.00)	1/16 (6.25)	.33
Mortality (*n*%)	4/15 (26.67)	0/15 (0)	<.01	1/16 (6.25)	0/16 (0)	.73

Recurrence rate and mortality refer to those 5 years after surgery.

Abbreviation: CAF, cancer-associated fibroblast; E, interior lesion stiffness of PTC patient; E_shell_, peri-cancerous tissue stiffness; LNM, lymph node metastasis; PTC, papillary thyroid carcinoma.

We further analyzed the association between CAF activation (αSMA, PDGFRα, and p-MLC2) and tissue stiffness in each patient with PTC subgroup. The results indicated that CAF activation was positively associated with lesion stiffness in both the LNM and non-LNM groups (all subgroups *r* ≥ 0.60 of αSMA, PDGFRα, and p-MLC2 with E and E_shell_; Figure 3E-J; all *P* < .001). Particularly noteworthy was the strong positive association observed with E_shell_/E (all subgroups *r* ≥ 0.80 of αSMA, PDGFRα, and p-MLC2 with E_shell_/E; Figure 3K-M; all *P* < .001).

### Different collagen contents and distributions in 4 subgroups of patients with PTC

**Figure 4.**
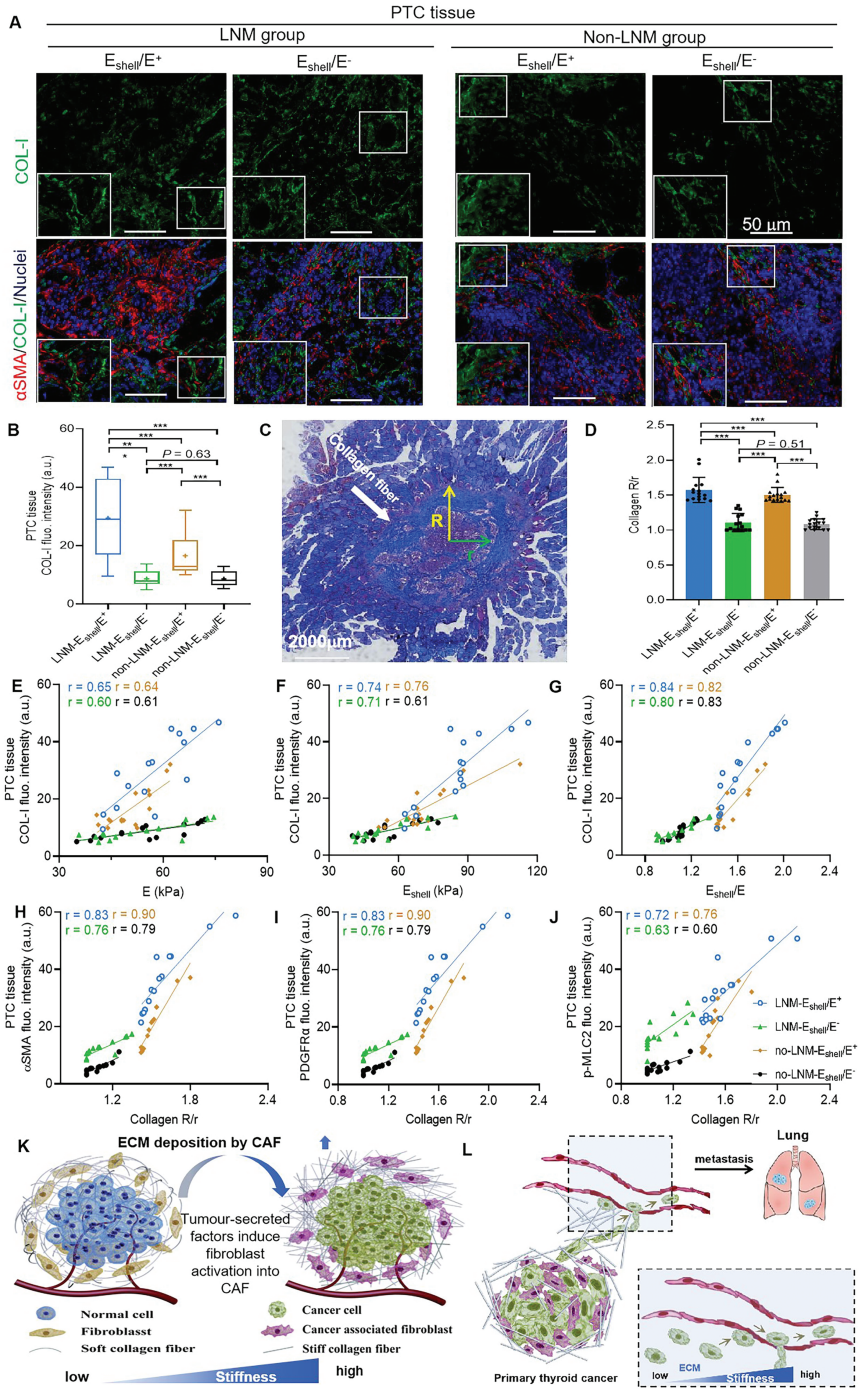
Different collagen contents and distributions in 4 subgroups of patients with PTC. (A) mIF staining of COL-I on paraffin sections of 4 subgroup patients. (B) Quantification of COL-I fluorescence intensity in 4 subgroups; that of the LNM-E_shell_/E^+^ group was significantly higher than that in the other 3 subgroups, and it was also higher in the non-LNM-E_shell_/E^+^ group than in the other 2 subgroups (all *P* < .001). (C) WSI image of thyroid cancer. The White arrow indicates the collagen fibers; *R* represents the distance from the center of thyroid cancer to the outer ring of collagen; *r* represents the distance from the center of thyroid cancer to the inner collagen ring. The higher the *R*/*r*, the more collagen fibers are deposited in the peri-cancerous tissues. (D) Quantification of collagen *R*/*r* of patients with PTC in 4 subgroups. The collagen *R*/*c* ratio of the E_shell_/E^+^ group was significantly higher than that of the E_shell_/E^−^ groups in patients with PTC, and the collagen *R*/*c* ratio in the LNM-E_shell_/E^+^ group was significantly higher than that in the other 3 subgroups (all *P* < .001). Pearson’s correlation analysis of the E value (E), E_shell_ value (F), and E_shell_/E (G) with fluorescence intensity of COL-I based on mIF in 4 subgroups of patient with PTC samples. The COL-I content in lesion samples of patients PTC in all subgroups was positively associated with tissue stiffness (all subgroups *r* ≥ 0.60 of COL-I with E and E_shell_, all *P* < .000), and especially highly positively associated with E_shell_/E (all subgroups *r* ≥ 0.80 of COL-I with E_shell_/E, all *P* < .001). Pearson’s correlation comparison analysis of collagen *R*/*r* ratio with fluorescence intensity of αSMA (H), PDGFRα (I), and p-MLC2 (J) based on mIF staining in 4 subgroups of patient with PTC samples. The collagen *R*/*c* ratio was positively associated with CAF activation (αSMA, PDGFRα, and p-MLC2) (all subgroups *r* ≥ 0.60 of αSMA, PDGFRα, and p-MLC2 with collagen *R*/*c* ratio, all *P* < .001). Schematic diagram of activated CAF stimulating collagen deposition and promoting PTC disease progression. (K) Normal cells are surrounded by normal fiber cells, secreting normal fibroblast, and the tissue stiffness is soft (left). MalignanT cells stimulate the activated CAFs, leading to collagen deposition in the peri-cancerous region, ECM remodeling, and an increase in tissue stiffness (right). (L) With heightened CAF activation in patients with PTC and an increase in collagen within the ECM, tissue stiffness rises, thus promoting malignanT-cell migration and further distant metastasis. In all panels, ***P < .001. Blue indicates the LNM-E_shell_/E^+^ group, *n* = 15. Green indicates the LNM-E_shell_/E^−^ group, *n* = 15. Brown indicates the non-LNM-E_shell_/E^+^ group, *n* = 16. Black indicates the non-LNM-E_shell_/E^−^ group, *n* = 16. Scale bars = 50 μm. Abbreviations: PTC, papillary thyroid carcinoma; COL-I, type 1 collagen; WSI, whole slide imaging; αSMA, alpha-smooth muscle actin; au, arbitrary unit; PDGFRα, platelet-derived growth factor receptor alpha; p-MLC2, phospho-myosin light chain 2; ECM, extracellular matrix; mIF, multiplex immunofluorescence.

The differences in COL-I content were analyzed in each subgroup of patient with PTC samples. The COL-I content in the samples of the LNM-E_shell_/E^+^ group was significantly higher than that in the other 3 subgroups, and it was higher in the non-LNM-E_shell_/E^+^ group than in the other 2 subgroups (all *P* < .001; Figure 4A and 4B). In the WSI sections of the 4 subgroups of patients with PTC, the ratio of the distance from the center of the cancer to the outer collagen ring (*R*) to the inner ring (*r*) in the peri-cancerous tissue was calculated (Figure 4C). The collagen *R*/*c* ratio of the E_shell_/E^+^ group was significantly higher than that of the E_shell_/E^−^ groups in patients with PTC, and the collagen *R*/*c* ratio in the LNM-E_shell_/E^+^ group was significantly higher than that in the other 3 subgroups (all *P* < .001; Figure 4D). The results suggested that collagen deposition in PTC patients in the E_shell_/E^+^ group mainly occurred in the peri-cancerous region especially in patients in the LNM-E_shell_/E^+^ subgroup.

In addition, COL-I content in lesion samples of patients with PTC in all subgroups was positively associated with tissue stiffness (all subgroups *r* ≥ 0.60 of COL-I with E and E_shell_; Figure 4E and F). Notably, there was a particularly strong positive association with E_shell_/E (all subgroups *r* ≥ 0.80 between COL-I and E_shell_/E; Figure 4G). We further analyzed the association between collagen *R*/*c* and CAF activation in subgroups of patients with PTC. In all subgroups of PTC patients, collagen R/c was positively associated with CAF activation markers (αSMA, PDGFRα, and p-MLC2) (all subgroups *r* ≥ 0.60 for αSMA, PDGFRα, and p-MLC2 with collagen *R*/*c*; Figure 4H-J). The results showed that the high activation of CAFs in the LNM-E_shell_/E^+^ subgroup resulted in collagen deposition in the peri-cancerous region and increased lesion invasiveness, which increased the probability of metastasis (Figure 4K-M).

### ECM remodeling and stiffening features in LNM-E_shell_/E^+^ group patients with PTC

**Figure 5.**
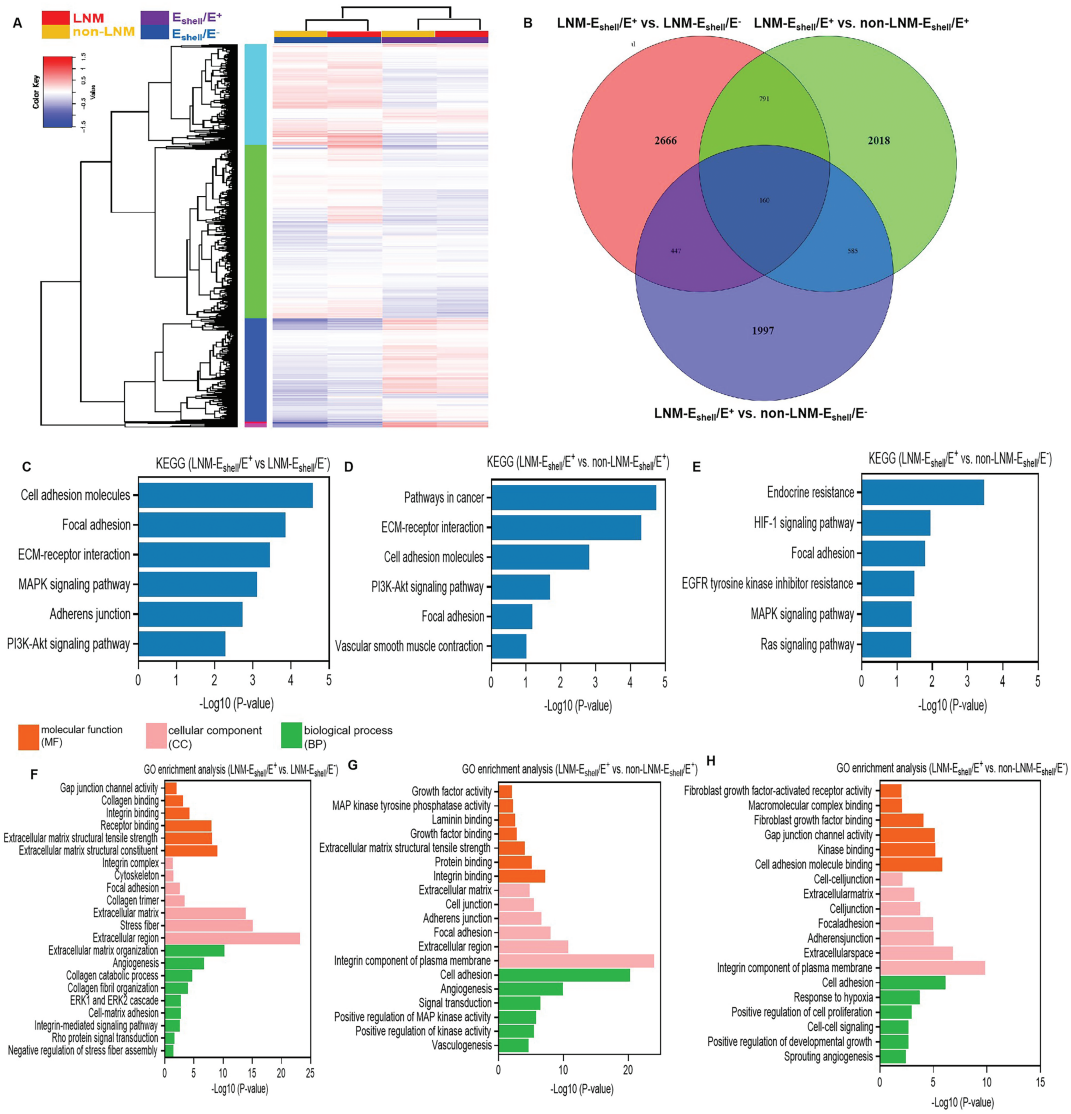
RNA-sequencing indicated that patients with PTC in the E_shell_/E^+^ group had multiple gene upregulation compared with those in the E_shell_/E^−^ group, suggesting ECM remodeling, fibrosis, enhanced cell motility, and increased migration ability in the LNM-E_shell_/E^+^ group. (A) Heatmap of differentially expressed genes in the E_shell_/E^+^ group (purple) and E_shell_/E^−^ group (blue). Red indicates high-expression genes, and blue indicates low-expression genes. The color ranges from blue to red, indicating higher gene levels in all subgroups [all subgroups, *n* = 5, each with absolute log2 (fold change) > 0.5 and FDR < 10%]. (B) Venn diagram presenting the number of differentially expressed genes specific to the LNM-E_shell_/E^+^ group compared with the other subgroups, as well as the number of differentially shared genes [2666 upregulated genes in LNM-E_shell_/E^+^ vs LNM-E_shell_/E^−^, 2018 genes upregulated in LNM-E_shell_/E^*+*^ vs non-LNM-E_shell_/E^+^, and 1997 genes upregulated in LNM-E_shell_/E^+^ vs non-LNM-E_shell_/E^−^; all subgroups, *n* = 5, all with an absolute log2 (fold change) > 0.5 and FDR < 10%]. (C-E) GSA of the top 1500 upregulated genes indicating comparisons between LNM-E_shell_/E^+^ vs LNM-E_shell_/E^−^, non-LNM-E_shell_/E^+^, and non-LNM-E_shell_/E^−^, revealing enriched KEGG pathways related to focal adhesion and ECM-receptor interaction. (F-H) GO pathway analysis revealed enrichment in ECM components, integrin-mediated pathways, and growth factor activity [all subgroups, *n* = 5; all with an absolute basemean > 10, *p*_adj_ < .01, and log2 (fold change) > 1]. Abbreviations: PTC, papillary thyroid carcinoma; ECM, extracellular matrix; LNM, lymph node metastasis; GSA, gene set analysis; KEGG, Kyoto Encyclopedia of genes and genomes; GO, gene ontology; FDR, false discovery rate.

The tissue samples of a total of 20 PTC patients (5 patients from each of the 4 subgroups) underwent RNA-seq. The clinical characteristics of these patients with PTC are presented in Table 4. patients with PTC in the E_shell_/E^+^ and E_shell_/E^−^ groups had differentially expressed genes (Figure 5A). Specifically, there were 2666 upregulated genes in LNM-E_shell_/E^+^ vs LNM-E_shell_/E^−^, 2018 upregulated genes in LNM-E_shell_/E^+^ vs non-LNM-E_shell_/E^+^, and 1997 upregulated genes in LNM-E_shell_/E^+^ vs non-LNM-E_shell_/E^−^ (Figure 5B). We compared the enriched upregulated pathways in 3 groups (LNM-E_shell_/E^+^ vs LNM-E_shell_/E^−^, LNM-E_shell_/E^+^ vs non-LNM-E_shell_/E^+^, and LNM-E_shell_/E^+^ vs non-LNM-E_shell_/E^−^) using GO (Figure 5C-E) and KEGG pathways (Figure 5F-H). KEGG analysis indicated that focal adhesion and ECM-receptor interaction-related genes were significantly upregulated in the LNM-E_shell_/E^+^ group. Focal adhesion indicates enhanced myofibroblast activity and cell motility. ECM-receptor interactions are critical for tissue fibrosis, the upregulation of which indicates ECM remodeling.^21^ GO identified upregulated ECM components, integrin-mediated pathways, and growth factor activity features in the PTC patients in the LNM-E_shell_/E^+^ group. Integrins are ECM receptors and function as mechanotransducers. Increased integrins in tumors promote malignancy by transducing ECM queues to cytoskeletal structures.^18^ All these results suggest that the LNM-E_shell_/E^+^ group PTC patients exhibited remarkable fibrosis and invasiveness features.

**Table 4.** Clinical characteristics of PTC patients participating in RNA sequencing.

Characteristics	LNM (*n* = 10)			Non-LNM (*n* = 10)		
	E_shell_/E^+^ (*n* = 5)	E_shell_/E^−^ (*n* = 5)	*p*	E_shell_/E^+^ (*n* = 5)	E_shell_/E^−^ (*n* = 5)	*p*
Sex (female *n*%)	3/5 (60.00)	3/5 (60.00)	1.00	9/16 (56.25)	9/16 (56.25)	1.00
Median age (IQR) Years	43 (21-50)	44 (34-60)	0.44	44 (26-64)	55 (35-66)	0.21
Age ≥ 60 years (*n*%)	3/5 (60.00)	3/5 (60.00)	1.00	5/5 (100.00)	5/5 (100.00)	1.00
Median tumor size (IQR) mm	15.8 (12.3-17.9)	17.1 (14.3-18.9)	0.44	16.7 (15.3-19.9)	18.8 (16.1-21.1)	0.21
Tumor size > 10 mm (*n*%)	5/5 (100.00)	5/5 (100.00)	1.00	5/5 (100.00)	5/5 (100.00)	1.00
Tumor stage III/IV	5/5 (100.00)	2/5 (40.00)	0.17	2/5 (40.00)	0/5 (0)	0.44
*BRAF* ^V600E^ mutation (*n*%)	4/5 (80.00)	4/5 (80.00)	1.00	4/5 (80.00)	4/5 (80.00)	1.00
^131^I treatment (*n*%)	5/5 (100.00)	5/5 (100.00)	1.00	2/5 (40.00)	2/5 (40.00)	1.00
Median follow-up time (months)	47 (24-60)	58 (55-59)	0.24	58 (55-60)	57 (56-60)	0.15
E (mean ± SD, kPa)	64.05 ± 4.12	64.33 ± 3.24	0.12	65.25 ± 3.13	65.45 ± 5.31	0.53
E_shell_ (mean ± SD, kPa)	91.28 ± 5.57	68.78 ± 5.64	< 0.001	78.72 ± 5.98	64.72 ± 4.62	< 0.001

Recurrence rate and mortality refer to those 5 years after surgery.

Abbreviations: E, interior lesion stiffness of PTC patient; E_shell_, peri-cancerous tissue stiffness.; LNM, lymph node metastasis; PTC, papillary thyroid carcinoma.

### CAF activation and Ki-67 expression increased in the lymph nodes of the E_shell_/E^+^ group in patients with PTC

**Figure 6.**
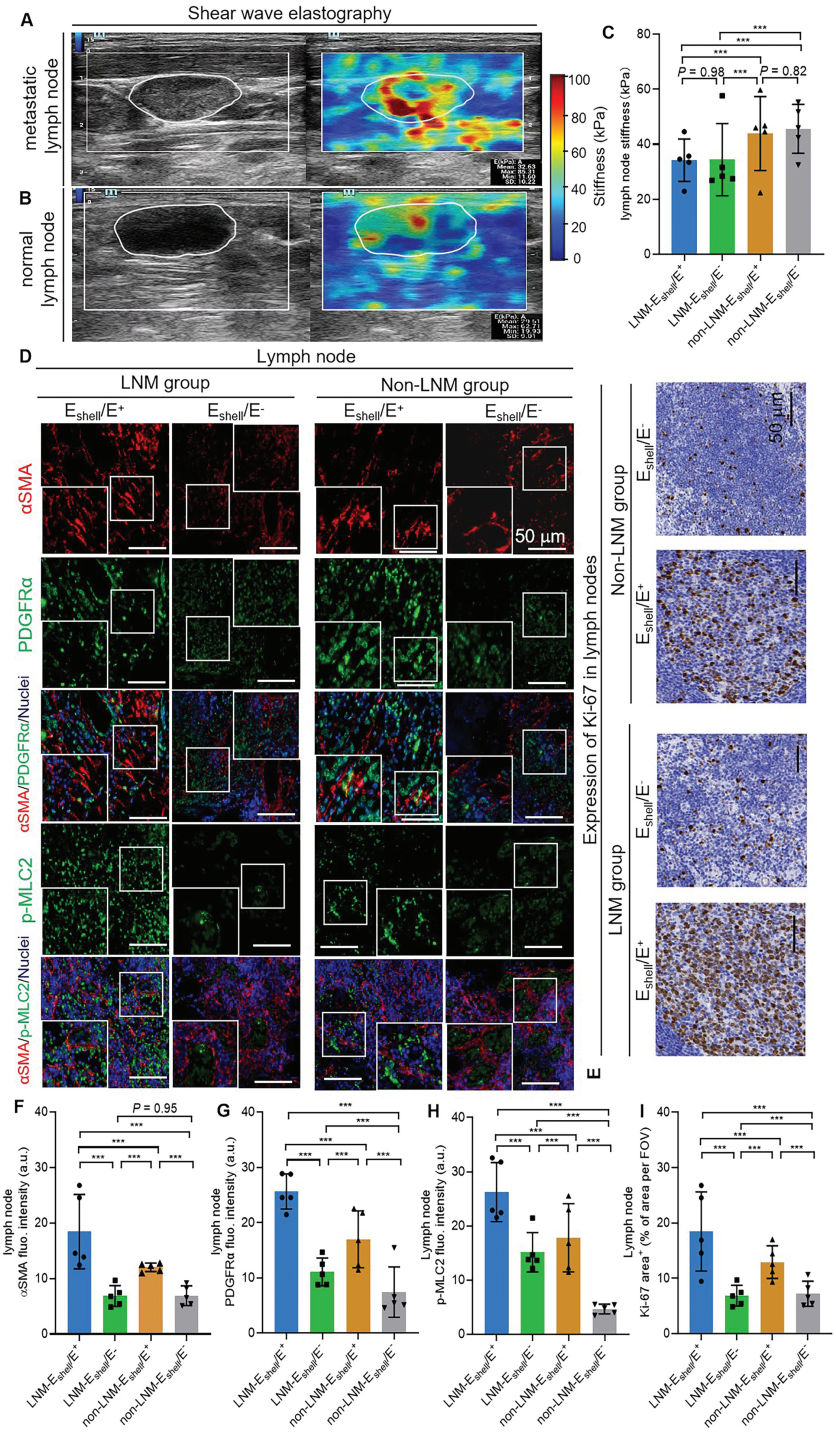
CAF activation and Ki-67 expression increased in the lymph nodes of E_shell_/E^+^ group patients with PTC. (A) SWE ultrasonography measured a tissue stiffness of 85.31 kPa in metastatic lymph nodes. (B) SWE ultrasonography measured a tissue stiffness of 62.71 kPa in normal lymph nodes. (C) Quantification of lymph node tissue stiffness in 4 subgroups of patients with PTC. There was no statistical difference in stiffness of lymph nodes between the LNM-E_shell_/E^+^ and LNM-E_shell_/E^−^ groups (34.22 ± 6.80 kPa vs 34.39 ± 2.89 kPa, *P* = .98), and the non-LNM-E_shell_/E^+^ and non-LNM-E_shell_/E^−^ groups (43.89 ± 7.20 kPa vs 45.59 ± 1.70 kPa, *P* = .82). (D) mIF staining of αSMA (red), PDGFRα (green), and p-MLC2 (green) on lymph node paraffin sections of patients of the 4 subgroups. (E) IHC positive area expression of Ki-67 on lymph node paraffin sections of patients of the 4 subgroups. (F-H) Quantification of (F) αSMA fluorescence intensity, (G) PDGFRα fluorescence intensity, and (H) p-MLC2 fluorescence intensity on lymph node paraffin sections based on mIF in 4 subgroups. A significantly higher expression of αSMA, PDGFRα, and p-MLC2 were observed in lymph nodes of the LNM-E_shell_/E^+^ group (all *P* < .001). (I) Quantification percentage of positive area expression of Ki-67 on lymph node paraffin sections from IHC in 4 subgroups. A significantly higher IHC positive area expression of Ki-67 was observed in lymph nodes of the LNM-E_shell_/E^+^ group (all *P* < .001). In all panels, *** *P* < .001. All subgroups, *n* = 5. Scale bars = 50 μm. Abbreviations: SWE, shear wave elastography; PTC, papillary thyroid carcinoma; αSMA, alpha-smooth muscle actin; au, arbitrary unit; FOV, field of view; PDGFRα, platelet-derived growth factor receptor alpha; p-MLC2, phospho-myosin light chain 2; COL-I, type 1 collagen; LNM, lymph node metastasis; IHC, immunohistochemical; mIF, multiplex immunofluorescence.

We included 5 patients for lymph node analysis from 4 subgroups of patients with PTC. The clinical characteristics of the study participants are presented in Table 5. There was no statistical difference in the stiffness of lymph nodes between the LNM-E_shell_/E^+^ and LNM-E_shell_/E^−^ groups (34.39 ± 2.89 kPa vs 34.22 ± 6.80 kPa, *P* = .98), or between the non-LNM-E_shell_/E^+^ and non-LNM-E_shell_/E^−^ groups (45.59 ± 1.70 kPa vs 43.89 ± 7.20 kPa, *P* = .82). However, the stiffness of lymph nodes in both LNM groups was lower than that in non-LNM lymph nodes, possibly due to the presence of microcystic lesions in the lymph nodes with metastasis (all *P* < .001; Figure 6A-C). We further analyzed the differences in CAF activation and Ki-67 content in lymph nodes among 4 subgroups of patients with PTC using IHC staining (Figure 6D). The lymph nodes of patients in the LNM-E_shell_/E^+^ group exhibited shared CAF activation features (αSMA, PDGFRα, and p-MLC2) similar to those of the tissue in the LNM-E_shell_/E^+^ group, as well as a higher IHC positive area expressing Ki-67 than those in other subgroups (all *P* < .001; Figure 6E-H).

**Table 5.** Clinical characteristics of PTC patients involved in the analysis of lymph node stiffness, CAF activation, and Ki-67 expression

Characteristics	LNM (*n* = 10)			Non-LNM (*n* = 10)		
	E_shell_/E^+^ (*n* = 5)	E_shell_/E^−^ (*n* = 5)	*P*	E_shell_/E^+^ (*n* = 5)	E_shell_/E^−^ (*n* = 5)	*p*
Sex (female *n*%)	3/5 (60.00)	3/5 (60.00)	1.00	9/16 (56.25)	9/16 (56.25)	1.00
Median age (IQR), years	43 (21-50)	44 (34-60)	.44	44 (26-64)	55 (35-66)	.21
Age ≥ 60 years (*n*%)	3/5 (60.00)	4/5 (80.00)	1.00	2/5 (40.00)	2/5 (40.00)	1.00
Median lymph nodes size (IQR), mm	12.8 (12.4-14.5)	13.6 (12.6-16.2)	.44	14.7 (12.3-18.6)	15.8 (13.3-17.9)	.29
Median thyroid cancer size (IQR), mm	11.9 (11.3-14.9)	12.5 (11.6-16.9)	.56	14.4 (12.3-17.6)	13.7 (12.4-15.9)	.33
Tumor stage III/IV (*n*%)	5/5 (100.00)	2/5 (40.00)	.17	2/5 (40.00)	0/5 (0)	.44
*BRAF* ^V600E^ mutation (*n*%)	4/5 (80.00)	4/5 (80.00)	1.00	4/5 (80.00)	4/5 (80.00)	1.00
^131^I treatment (*n*%)	5/5 (100.00)	5/5 (100.00)	1.00	1/5 (20.00)	1/5 (20.00)	1.00
Median follow-up time (months)	47 (24-60)	58 (55-59)	.24	58 (55-60)	57 (56-60)	.15
E (mean ± SD, kPa)	34.22 ± 6.80	34.39 ± 2.89	.98	43.89 ± 7.20	45.59 ± 1.70	.82

Abbreviations. E, lymph node stiffness; LNM, lymph node metastasis; PTC, papillary thyroid carcinoma.

## Discussion

PTC is the most common pathological type of thyroid cancer, and it has the best prognosis.^1^ However, the impact of LNM on the prognosis of PTC has been controversial.^2-8^ While some studies suggest that LNM predicts a poor prognosis for patients with PTC, others disagree. For example, in childhood PTC, the probability of LNM occurrence is high, at 90%, yet the prognosis remains favorable.^9^ Previous studies often focused solely on the presence or absence of LNM without further analyzing the difference in risk associated with LNM.^2-8^ There may be more factors contributing to the different types of prognosis of patients with PTC with LNM. Due to inconsistent reports on the role of cervical LNM in the clinical outcomes of PTC, we seized the opportunity presented by this large multicenter cohort to conduct a comprehensive investigation. Our study found that the peri-cancerous tissue stiffness states are an important factor affecting LNM-related aggressiveness in patients with PTC.

Our results revealed a significantly higher UE area/US area ratio in the thyroid lesion of the LNM group than in that of the non-LNM group. The E_shell_ and E_shell_/E values in the LNM group were significantly higher than those in the non-LNM group; however, there was no statistical difference in the E value (Figure 2A-F). This may indicate that the tissue stiffness associated with the LNM-related aggressiveness in PTC is derived from the peri-cancerous tissue rather than the interior cancer. This is similar to previous conclusions that peri-cancerous tissue stiffness is more effective than the interior stiffness of cancer in differentiating between benign and malignant thyroid nodules.^12-16^ Similar findings have been reported in studies of tumors in other organs, such as malignant breast lesions. The presence of highly stiff tissue surrounding breast lesions is indicative of increased aggressiveness, commonly referred to as the “stiff rim” sign.^20,22^ Through QUEST classification tree detection,^16^ when the E_shell_/E ratio was less than or equal to 1.412, regardless of the presence of LNM, the probability of postoperative recurrence in patients with PTC was only 2.60%. We were pleasantly surprised to find that in patients with PTC with both E_shell_/E > 1.412 and LNM, the probability of postoperative recurrence in patients with PTC was as high as 91.60% (Figure 2I). Thus, the E_shell_/E ratio could be used to differentiate the prognosis of patients with PTC with LNM into 2 different outcomes. While LNM is a related but not independent factor affecting the prognosis of patients with PTC, our results indicated that after further grouping LNM group patients based on tissue stiffness, the recurrence, and mortality rate of patients with PTC in the LNM-E_shell_/E^+^ group were significantly increased compared with those in the original LNM group. In contrast, the recurrence rate and mortality in the LNM-E_shell_/E^−^ group were significantly lower than those in the original LNM group (Figure 2K-L, all *P* < .001). By further grouping patients based on E_shell_/E, the risk of LNM-related mortality in patients with PTC was divided into 2 different results (LNM E_shell_/E^+^: 7.29% vs LNM E_shell_/E^−^: 0.22%, *P* < .001, Table 2), illustrating the inconsistency observed in previous studies when considering LNM as a single factor in the risk of mortality in PTC patients.^2-7^

The fluorescence intensity of αSMA, PDGFRα, p-MLC2, and COL-I based on mIF staining in the LNM-E_shell_/E^+^ and non-LNM-E_shell_/E^+^ groups was significantly higher than that in the other 2 subgroups (Figures 3A-D and 4A and B, all *P* < .001). Furthermore, the expression of αSMA, PDGFRα, p-MLC2, and COL-I in each subgroup was positively associated with the E value, E_shell_ value, and E_shell_/E of patients with PTC (all *r* > 0.60) and highly positively associated with E_shell_/E (all *r* > 0.80) (Figures 3E-M and 4E-G, all *P* < .001). This explains why the recurrence rate and mortality of PTC patients in the E_shell_/E^+^ groups were significantly higher than those in the E_shell_/E^−^ groups. The collagen *R*/*r* ratio in the LNM-E_shell_/E^+^ group was also significantly higher than that in patients with PTC in the other 3 subgroups (Figure 4C and D). A wider collagen R/r indicates a more extensive distribution of collagen fibers in the peri-cancerous tissue, resulting in increased stiffness and a higher degree of cancer aggressiveness. The collagen *R*/*r* ratio was also positively associated with αSMA, PDGFRα, p-MLC2, the E value, the E_shell_ value, and the E_shell_/E ratio of patients with PTC in all subgroups (Figure 4H-J, all *P* < .001). These results not only prove that the perinodular tissue stiffness can more accurately identify benign and malignant thyroid nodules than the interior tissue stiffness, but also further suggest the role of peri-cancerous tissue stiffness state in predicting LNM-related invasiveness in patients with PTC.^11-15^ There was a further interesting phenomenon present in our results: although the recurrence rate, mortality, CAF activation, and COL-I content of patients with PTC in the non-LNM-E_shell_/E^+^ group were lower than those in the LNM-E_shell_/E^+^ group, they were all higher than those in 2 E_shell_/E^−^ groups. There are 2 possible reasons for this. First, patients with PTC with LNM were not found to have positive lymph nodes on pathological examination, resulting in patients originally belonging to the LNM-E_shell_/E^+^ group and non-LNM-E_shell_/E^+^ group. Second, patients with PTC in the non-LNM-E_shell_/E^+^ group were in an earlier stage compared to those in the LNM-E_shell_/E^+^ group. In other words, patients in the non-LNM group had not yet developed LNM, although it may have occurred later. These results are important for determining surgical strategies for PTC patients, which may be helpful for improving their quality of life and reducing their mortality rate.

RNA-seq revealed that, compared to patients with PTC in the other 3 subgroups, those in the LNM-E_shell_/E^+^ group exhibited upregulation of multiple genes associated with activated myofibroblasts, enhanced cell adhesion, increased fibrosis, and ECM remodeling (Figure 5A-H). These results suggested that the LNM-E_shell_/E^+^ group patients with PTC had a poor prognosis. Previous similar results suggested that activated fibroblasts may correlate with advanced disease and worse prognosis, as observed in other tumors.^18,21,23,24^

To further verify that the E_shell_/E ratio increases LNM-related invasiveness in patients with PTC, we quantitatively analyzed CAF activation and Ki-67 expression in the lymph nodes of 4 subgroups of patients with PTC. Our results indicated that CAF activation and Ki-67 expression in the lymph nodes of the LNM-E_shell_/E^+^ group were significantly higher than those in the other subgroups (Figure 6A-H, all *P* < .001). These findings suggested that there was more active lymph node cell proliferation in the LNM-E_shell_/E^+^ group than in the other groups, and also that the possibility of distant metastasis was higher, both of which are consistent with our previous results.

Our study has several limitations that should be noted. (1) The analysis involved a limited number of patients for both cancerous tissue samples and lymph node tissue samples. (2) The selection of patients for lymph node tissue sample analysis was based on ultrasound imaging that identified suspicious lymph nodes. This approach has a certain rate of missed diagnoses, as some pathologically metastatic lymph nodes may be overlooked due to atypical ultrasound features, resulting in the loss of ultrasound elasticity data. However, the analysis of lymph node tissue samples provides corroborative evidence for the biological information related to the stiffness of cancerous and peri-cancerous tissues in patients with PTC. Our study’s focus remains on the stiffness of the cancerous and peri-cancerous tissues in patients with PTC. We plan to increase the number of patients participating in lymph node tissue sample analysis in future studies to more accurately reflect the biological state of these tissues in patients with PTC. (3) The E_shell_/E > 1.412 threshold obtained in our study was based on research using the Mindray Resona 7 ultrasonography device. The variability in technologies used by different ultrasound elastography devices limits the applicability of this threshold across various devices.

Despite these limitations, our study offers valuable insights into the role of peri-cancerous tissue stiffness in the prognosis of patients with PTC. Our results suggest that when patients with PTC have both LNM and E_shell_/E > 1.412, more aggressive surgical approaches and close follow-up protocols should be adopted. Even in the absence of LNM, when E_shell_/E > 1.412 alone is present, a more proactive treatment model, such as radioactive iodine therapy, should be considered. This is because our results indicate that patients with PTC without LNM but with E_shell_/E > 1.412 are also at risk for distant metastasis. Furthermore,research with larger sample sizes, standardized lymph node evaluation methods, and a variety of ultrasound elastography devices is required to validate our findings and explore the underlying biological mechanisms in greater detail.

## Conclusion

Our study provides compelling evidence that peri-cancerous stiffness serves as a critical biomarker for stratifying the LNM-related recurrence rate and mortality in PTC patients into distinct prognostic categories. Specifically, an E_shell_/E ratio greater than 1.412 emerges as a pivotal threshold, signifying heightened CAF activation. This activation is intricately linked with extensive ECM remodeling and subsequent tissue fibrosis, which collectively facilitate malignanT-cell metastasis. The association of this biomechanical parameter with an escalated LNM-related recurrence rate and mortality underscores its potential utility in clinical practice. By adopting this novel marker, clinicians can more accurately predict disease progression, enabling the implementation of tailored therapeutic strategies aimed at mitigating the risk of metastasis and improving patient outcomes in PTC. This study, therefore, lays the groundwork for integrating biomechanical insights into the prognostic evaluation of thyroid cancer, offering a promising avenue for enhancing personalized medicine in oncology.

## Data Availability

The raw data supporting the conclusions of this article will be made available by the authors. Furthermore,inquiries can be directed to the first author, Lei Hu (hulei@ustc.edu.cn).
