# Correction to: Enhanced stiffness in peri-cancerous tissue: a marker of poor prognosis in papillary thyroid carcinoma with lymph node metastasis

**DOI:** 10.1093/oncolo/oyae208

**Published:** 2024-08-13

This is a correction to: Lei Hu, Lei Ye, Chong Pei, Chunlei Sun, Chaoxue Zhang, Fan Jiang, Nianan He, Weifu Lv, Enhanced stiffness in peri-cancerous tissue: a marker of poor prognosis in papillary thyroid carcinoma with lymph node metastasis, The Oncologist, 2024;, oyae086, https://doi.org/10.1093/oncolo/oyae086

In the originally published version of this manuscript, the symbol indicating equal first authorship of authors Lei Hu, Lei Ye and Chong Pei was omitted from the author byline.

The Publisher apologizes for this error, which has been corrected.

